# Stakeholder Perspectives on the Development of a Virtual Clinic for Diabetes Care: Qualitative Study

**DOI:** 10.2196/jmir.9.3.e23

**Published:** 2007-08-09

**Authors:** Natalie Armstrong, Hilary Hearnshaw, John Powell, Jeremy Dale

**Affiliations:** Health Sciences Research InstituteWarwick Medical School, University of WarwickCoventryUK

**Keywords:** Virtual clinic, diabetes, Internet, stakeholder consultation, consumer health informatics, focus groups

## Abstract

**Background:**

The development of the Internet has created new opportunities for health care provision, including its use as a tool to aid the self-management of chronic conditions. We studied stakeholder reactions to an Internet-based “virtual clinic,” which would allow people with diabetes to communicate with their health care providers, find information about their condition, and share information and support with other users.

**Objective:**

The aim of the study was to present the results of a detailed consultation with a variety of stakeholder groups in order to identify what they regard as the desirable, important, and feasible characteristics of an Internet-based intervention to aid diabetes self-management.

**Methods:**

Three focus groups were conducted with 12 people with type 1 diabetes who used insulin pumps. Participants were recruited through a local diabetes clinic. One-on-one interviews were conducted with 5 health care professionals from the same clinic (2 doctors, 2 nurses, 1 dietitian) and with 1 representative of an insulin pump company. We gathered patient consensus via email on the important and useful features of Internet-based systems used for other chronic conditions (asthma, epilepsy, myalgic encephalopathy, mental health problems). A workshop to gather expert consensus on the use of information technology to improve the care of young people with diabetes was organized.

**Results:**

Stakeholder groups identified the following important characteristics of an Internet-based virtual clinic: being grounded on personal needs rather than only providing general information; having the facility to communicate with, and learn from, peers; providing information on the latest developments and news in diabetes; being quick and easy to use. This paper discusses these characteristics in light of a review of the relevant literature. The development of a virtual clinic for diabetes that embodies these principles, and that is based on self-efficacy theory, is described.

**Conclusions:**

Involvement of stakeholders is vital early in the development of a complex intervention. Stakeholders have clear and relevant views on what a virtual clinic system should provide, and these views can be captured and synthesized with relative ease. This work has led to the design of a system that is able to meet user needs and is currently being evaluated in a pilot study.

## Introduction

The development of the Internet has created new opportunities for health care provision, including its use as a tool to aid the self-management of chronic conditions. In the United Kingdom, there are over 2 million people diagnosed with diabetes, and Internet-based interventions may represent a way of helping them self-manage their condition from home. There have been several recent studies of Internet-based interventions with patients carried out in Europe [1-4] and North America [5-8]. A report of a small study that pilot-tested the feasibility of allowing patients with type 2 diabetes to co-manage their condition from home [9] showed proof of concept. The virtual clinic concept studied here is such a system, aimed at people receiving care for diabetes from the UK National Health Service (NHS), and it is enhanced by being based on the behavioral theory of self-efficacy [10]. The theory suggests that to enhance self-efficacy (the confidence an individual has that he or she can achieve a particular objective) an intervention should increase autonomy, reduce negative perceptions of being different, offer vicarious learning and modelling opportunities from peers, encourage setting of achievable goals, and give rewards for such achievements. Enhanced self-efficacy, in turn, increases the implementation of successful self-management. A systematic literature review of behavioral interventions for adolescents with type 1 diabetes found that those interventions that were theoretically based were significantly more effective than those that were not [11].

The aim of this research was to consult stakeholder groups and explore their perspectives on the desirable, important, and feasible characteristics of an Internet-based virtual clinic system for people who have type 1 diabetes and use insulin pumps, and to flag any potential worries or concerns. This builds on a recently conducted survey of potential users [12]. Gathering stakeholder perspectives is a vital first stage in order to ensure that the system developed meets the needs of future users [13] and that it achieves its full potential in their eyes [14]. Currently, much of the work to gather user feedback takes place following development of the system in question. In contrast, the objective of the study reported here was to undertake detailed consultation with a variety of stakeholder groups and feed their comments directly into the process of developing the system. It was envisaged that the virtual clinic would provide people living with diabetes, and their health professionals, access to the records of their condition (including uploaded blood glucose readings), a messaging facility, information and advice for patients, and a peer-to-peer support area, thereby meeting many of the criteria identified as desirable within Internet-based diabetes management [15].

## Methods

In order to gather detailed comments from the stakeholder groups identified, a qualitative approach was taken. Several elements were used, including focus groups, individual interviews, email consensus gathering, and an expert workshop.

Participants in both the focus groups and interviews received an information sheet about the study and gave their written consent to take part. The elements of the study were approved by an NHS Local Research Ethics Committee.

### Focus Groups

Three focus groups were held with people who had type 1 diabetes and who used insulin pumps to control their disease. These participants were recruited from a local diabetes clinic. Each focus group had between 3 and 5 participants. The total number of participants was 12 (2 male and 10 female), and all were over 16 years of age. All participants had responded to a written invitation to take part in the research, indicating at least a basic level of literacy. All those who responded to our written invitations were subsequently asked to take part in one of the focus groups. Although focus groups were held during the day and evening, a small number of those willing to take part could not attend any of the sessions. These people were invited to submit their views by email, which were included in the email consensus discussed below. Focus groups were conducted at the education center within the hospital at which the clinic was based, a convenient and familiar location. No health professionals were present at the focus group sessions. At each session, a short demonstration was given to familiarize participants with the concepts we were planning to develop. This comprised the following elements: (1) a demonstration of an existing system that could record physiological data (such as blood glucose readings) and facilitate patient/health professional messaging, and (2) a description of some additional features we thought may be of interest to potential users, including downloadable information, links to other relevant websites, and peer-to-peer discussion boards. The facilitator then used the topic guide to focus the discussion on the following: participants’ initial reactions to the concept, the most and least important/useful elements, whether they would be likely to use such a system, factors that may facilitate or hinder use, what the benefits may be, and any concerns they may have. Sessions were audio-recorded and lasted an average of 1 hour.

### Individual Interviews

Individual interviews were carried out with 5 health professionals from the same clinic (2 doctors, 2 specialist nurses, 1 dietician) and with 1 representative from an insulin pump manufacturer/supplier who had emerged from the focus group discussions as an important source of information and support. The same demonstration was given as for the focus groups, and the interviews included similar questions. All interviews were audio-recorded and lasted between 30 minutes and 1 hour.

### Consensus Gathering by Email

A process of email consensus gathering was carried out with patients using Internet-based systems for other chronic conditions in order to gain their views on the potential of the virtual clinic and their experience of using other systems. A message was posted on discussion boards aimed at patients with long-term conditions, including asthma, epilepsy, and mental health problems, and also on a discussion board for insulin pump users in order to consult further with the target group for the intervention being developed. Users were invited to contribute their views by email.

### Expert Workshop

An expert workshop was held at which invited delegates from the United Kingdom discussed the role of information technology (IT) in diabetes care. Delegates had a range of backgrounds, with expertise in areas such as diabetes management, the use of IT in health care, and patient involvement in long-term conditions. They included academic researchers (with backgrounds as diverse as primary care, psychology, psychiatry, engineering, and sociology), diabetologists, people living with diabetes, and a representative from the charity Diabetes UK. Delegates discussed the proposed system in small groups and completed questionnaires addressing the same issues as in the focus groups and interviews.

### Data Analysis

Qualitative data analysis was undertaken by the first and second author. Focus group and interview transcripts, email responses from users of other systems, and the questionnaires completed during the expert workshop were all preliminarily analyzed independently by each author using a thematic analysis approach. Recurrent themes were identified as they emerged from the data, rather than on the basis of researcher preconceptions. Following this, the two authors held an analysis meeting at which the emergent themes identified by each were compared and discussed. Following agreement on these, each author undertook a full analysis of approximately half the transcripts, with a small overlap to allow comparison of theme interpretation and allocation of data extracts to particular themes.

## Results

Six key themes, some with subthemes, were identified from the data and are described in detail below:

1. communication between patients and health professionals
2. presentation of patient data and permanency of the record
3. the importance and value of peer support
4. awareness of the personal nature of diabetes
5. how an Internet-based system would fit with the current provision of care
6. an Internet-based system may not be suitable for all people with diabetes

### Communication Between Patients and Health Professionals

The facility to send and receive secure messages through an Internet-based system was largely welcomed by all the stakeholder groups consulted, although there were some concerns. For health professionals, one of the key benefits was that the means through which they communicated with their patients outside of their face-to-face appointments would be more standardized. Having an asynchronous messaging system was seen to be beneficial as both parties could check their messages when it was convenient for them and fit this into their other activities, thereby saving a lot of time. This concurs with previous research [16,17]. For example, one health professional (HP) illustrated the time taken in trying to respond to a patient enquiry that had been left on the clinic answer phone:

HP6 I rang somebody and she said, “I’m right in the middle of shopping in [town]. Can you ring me back?” So, there’s a lot of time that you spend sort of ringing them. Or, I’ve got a school teacher, and she’s rang four times today and I have rang her back several times, and you know eventually [that] you get to talk but it might be a couple of days, so I think quite a lot of our patients feel that it’s easier to email us.

Similarly, a focus group participant (FGP) explained how she had largely given up trying to seek advice through the clinic helpline as she found having to leave a message and waiting to be called back simply unworkable:

FGP3 It’s so hard.... You have to leave a message, they ring you back, you’re in an open office so you can’t talk, so you just bash your way through it and hope it’s going to come out right at the end.

Being able to send a message and receive a reply was therefore welcomed by patients, so long as the system was adequately supported and health professionals responded to messages in a timely manner [18]. There were potential concerns raised by some health professionals that the number of patient contacts would increase and that the workload of the consultants would similarly increase since most patient messages had previously been filtered by the specialist nurses. These were fairly minor concerns though and did not outweigh the perceived benefits.

### Presentation of Patient Data and Permanency of the Record

One of the key advantages of the proposed Internet-based system identified by health professionals was the benefit of having ready access to a patient’s blood glucose readings. The advantages were two-fold: (1) having up-to-date results readily accessible, and (2) having results in a standard format. At present, health professionals were often faced with results in various formats:

HP2 Some of them [records kept by patients] might be too scribbled and not really very clear.... But, yes, scraps of paper might have, sometimes, [the time of day]...written in the corner, and you’re trying to see the time of the day and sort of trying to make them in your head into [a] date profile to some extent. So, they can be a bit difficult, yes.

The permanency of the record was also seen as beneficial by health professionals since, in contrast to a paper diary, the readings would not leave with the patient and would still be available for review and reflection at a later date. The potential for use as a teaching and training tool was also raised. In contrast, patients were more divided on the benefits of uploading their results. Those who found recording their readings problematic welcomed anything that would make it easier and more convenient, but for others the system was seen to offer nothing new. However, all patients recognized the benefit of the health professional having access to their readings when dealing with queries.

### The Importance and Value of Peer Support

In common with other research [5,19,20], one of the most valuable elements of the system identified by patients, and also recognized by health professionals, was peer-to-peer support. Patients in particular identified two key ways in which such support would be useful. First, being able to pick up tips and suggestions for managing their diabetes was viewed as beneficial, even for those who had had diabetes for some time:

FGP6 But there are so many things that you can come across for the first time, and the one I had to seek advice on was the flu injection last year, which caused chaos, and I thought, “Well, is this the flu injection or is there something else that I’m missing?”.... But again, a [discussion] board like this, just to push the question in and see what response you get back...

Second, being able to communicate with someone who understood what they were going through was considered very valuable by patients, particularly for those who did not know others with diabetes, thereby demonstrating the potential of Internet-based communication to move beyond the individual’s usual sphere of contacts.

FGP9 Within my life, around work, around home, and around socializing, I know no one else with diabetes, so to be able to get on...[the] Internet and sort of like to be able to have a chat with somebody... Even like you say, sometimes it might just be for a moan, but sometimes you can get so frustrated with it that you just want to be able to take it all out.... You want to be able to talk to someone, but finding... You talk to your partner and you talk to your friends, but they don’t always understand what you’re saying.

The health professionals also recognized the potential benefits of peer-to-peer support for their patients, particularly the ways in which Internet-based provision extended the group sessions currently offered at the clinic.

HP1 We have group sessions...and they are teaching each other...from their experience.... And they’ll be able to do that on a regular basis, on a daily basis, rather than on a three-monthly basis, and they’ll all be there in the chat room, potentially, rather than just the people who turned up to the group clinic.

However, there were some minor concerns among health professionals that the peer-to-peer support could lead to the “propagation of myths” and to individuals passing on what had worked for them to others for whom it may be inappropriate. It was generally felt that monitoring the topics under discussion would be sufficient to counter these concerns. The issue of patients’ awareness and appreciation of the individual nature of diabetes is discussed below. A recent systematic review of the effects of online peer-to-peer interactions [21] found no evidence to support concerns of causing harm, although it also failed to find robust evidence of any health benefit.

### Awareness of the Personal Nature of Diabetes

As stated above, one of the concerns health professionals had about the peer-to-peer support was that patients using it may not realize that what had worked for one person may not work for others:

HP1 Patients frequently want to propagate what has been good for them, and it might not be good for everybody, if you know what I’m saying. They might have found some particular way of dealing with situations...but it doesn’t always apply to everybody.

However, it was clear from the focus groups with patients that they were very well aware of how diabetes affected them personally and that this may differ markedly between individuals. The knowledge and experience they had built up from managing their diabetes on a day-to-day basis were substantial, and they spoke at length about the need to evaluate suggestions posted on discussion boards in the light of their personal situation. The following is a typical example:

FGP8 Whatever I read on there [the discussion board] may be useful, but I know it’s not individually designed for me. So, overall it can be a useful guide when you start out, but you really do have to know your own system, don’t you.

This ability on the part of potential users to appraise the information posted on a discussion board and evaluate whether it is likely to be useful to them, together with existing research demonstrating that most information posted on boards of this kind is accurate (or very quickly corrected by other participants) [22], indicates that the concerns voiced by health professionals are likely to be largely unfounded.

### How an Internet-Based System Would Fit With the Current Provision of Care

The general consensus among the focus group participants and health professionals was that the proposed Internet-based system would fit well with existing clinic provision. The system would provide a useful means of communication and support between routine clinic appointments and would bring benefits in terms of increased standardization, more efficient means of communication, and extended scope of current group consultations through the use of Internet-based asynchronous discussion boards and real-time chat rooms. Some concerns were raised about a potential increase in the demand of health professionals’ time for dealing with messages received from patients, and this was identified as a potential factor that could hinder usage. However, the general consensus was that, in the local clinic at least, any additional time would be minimal and may well be offset through the time savings brought about by other benefits. This is one of the issues to be explored in the planned pilot study.

### An Internet-Based System May Not Be Suitable for All

The vast majority of participants in the stakeholder consultation raised the point that an Internet-based system of the type proposed would not be suitable or appropriate for all people with diabetes. The main issue centered on the need for potential users to be comfortable with the required technology and to have a computer with Internet access. It was suggested that this may limit potential users to those who are younger and more familiar with technology, as supported by research by Giménez-Pérez et al [23]. However, a recent study by McKay et al [6] has shown that novice computer users will participate in an Internet-based system to assist their self-management. A further concern, raised particularly by a health professional, was that the system could be unsuitable for those with a poor understanding of their condition or for whom diabetes is only one of many health problems.

## Discussion

The research was successful in consulting stakeholder groups to explore their perspectives on the desirable, important, and feasible characteristics of an Internet-based system for diabetes. Involvement of stakeholders is a vital early stage in the development of a complex intervention, yet all too often their views are only gathered in later stages. Stakeholders have clear and relevant views on what such a system should provide, and these views can be captured and synthesized with relative ease. In this case, we identified 6 themes that are supported by findings from previous studies on the perceived convenience of an asynchronous messaging system [16,17] and the importance and value of peer-to-peer support [5,19,20]. The themes also contribute to debate about who is likely to use Internet-based systems and suggest that patients are eminently capable of assessing advice posted by peers on a discussion board and relating it to their personal situation. By basing the focus groups and interviews in an existing diabetes clinic, the research was able to suggest that an Internet-based system is likely to fit well with existing care provision as well as to explore the likely impact on health professionals’ time.

The main strength of this study is the involvement of a diverse range of stakeholders in detailed consultation during the development stage of an Internet-based intervention. The diverse range of methods used to involve these stakeholders (ie, interviews, focus groups, email consensus gathering, and an expert workshop) is also a key strength. In particular, the use of focus groups and a workshop means that we allowed participants to discuss and develop each others’ perspectives. However, there are also some limitations to the study. Patients were self-selecting volunteers, so they may have different views from those who did not volunteer to participate. It is noticeable, for example, that more women than men volunteered to take part. There is also a potential issue concerning how the perspectives of patients using insulin pumps relate to those of patients who control their type 1 diabetes through other means. We are aware that much of the feedback from patients does not specifically relate to their use of an insulin pump; rather, it is concerned with more generic issues such as communication with their health professionals and the value placed on peer support. However, we acknowledge that further stakeholder consultation with the target group(s) would be necessary before the intervention could be developed for diabetes patients who do not use insulin pumps.

This consultation demonstrated that an Internet-based system is attractive to the stakeholders consulted during the course of this study and has led to the design of a system that is able to meet their needs. This system has now been developed and is being evaluated in a pilot study.

## Multimedia Appendix

Presentation of this research (ppt)
						

## Abbreviations

FGP: focus group participant
HP: health professional
IT: information technology
NHS: National Health Service

## References

[1] Ferrer-Roca O, Franco Burbano K, Cárdenas A, Pulido P, Diaz-Cardama A (2004). Web-based diabetes control. J Telemed Telecare.

[2] Bellazzi R, Larizza C, Montani S, Riva A, Stefanelli M, D'Annunzio G, Lorini R, Gomez E J, Hernando E, Brugues E, Cermeno J, Corcoy R, De Leiva A, Cobelli C, Nucci G, Del Prato S, Maran A, Kilkki E, Tuominen J (2002). A telemedicine support for diabetes management: the T-IDDM project. Comput Methods Programs Biomed.

[3] Gómez E J, Hernando M E, García A, Del Pozo F, Cermeño J, Corcoy R, Brugués E, De Leiva A (2002). Telemedicine as a tool for intensive management of diabetes: the DIABTel experience. Comput Methods Programs Biomed.

[4] Cavan D A, Everett J, Plougmann S, Hejlesen O K (2003). Use of the Internet to optimize self-management of type 1 diabetes: preliminary experience with DiasNet. J Telemed Telecare.

[5] McKay H G, Feil E G, Glasgow R E, Brown J E (1998). Feasibility and use of an Internet support service for diabetes self-management. Diabetes Educ.

[6] McKay H G, Glasgow R E, Feil E G, Boles S M, Barrera M (2002). Internet-based diabetes self-management and support: initial outcomes from the diabetes network project. Rehabil Psychol.

[7] Glasgow Russell E, Boles Shawn M, Mckay H Garth, Feil Edward G, Barrera Manuel (2003). The D-Net diabetes self-management program: long-term implementation, outcomes, and generalization results. Prev Med.

[8] McMahon Graham T, Gomes Helen E, Hickson Hohne Sara, Hu Tang Ming-Jye, Levine Betty A, Conlin Paul R (2005). Web-based care management in patients with poorly controlled diabetes. Diabetes Care.

[9] Goldberg Harold I, Ralston James D, Hirsch Irl B, Hoath James I, Ahmed Kazi I (2003). Using an Internet comanagement module to improve the quality of chronic disease care. Jt Comm J Qual Saf.

[10] Bandura A (1977). Self-efficacy: toward a unifying theory of behavioral change. Psychol Rev.

[11] Hampson S E, Skinner T C, Hart J, Storey L, Gage H, Foxcroft D, Kimber A, Cradock S, McEvilly E A (2000). Behavioral interventions for adolescents with type 1 diabetes: how effective are they?. Diabetes Care.

[12] Lowe Pam, Hearnshaw Hilary, Griffiths Frances (2005). Attitudes of young people with diabetes to an Internet-based virtual clinic. J Telemed Telecare.

[13] Ralston James D, Revere Debra, Robins Lynne S, Goldberg Harold I (2004). Patients' experience with a diabetes support programme based on an interactive electronic medical record: qualitative study. BMJ.

[14] Kerr Cicely, Murray Elizabeth, Stevenson Fiona, Gore Charles, Nazareth Irwin (2006). Internet interventions for long-term conditions: patient and caregiver quality criteria. J Med Internet Res.

[15] Mazzi Christian P, Kidd Michael (2002). A framework for the evaluation of Internet-based diabetes management. J Med Internet Res.

[16] Tjora Aksel, Tran Trung, Faxvaag Arild (2005). Privacy vs usability: a qualitative exploration of patients' experiences with secure Internet communication with their general practitioner. J Med Internet Res.

[17] Lin Chen-Tan, Wittevrongel Loretta, Moore Laurie, Beaty Brenda L, Ross Stephen E (2005). An Internet-based patient-provider communication system: randomized controlled trial. J Med Internet Res.

[18] Car Josip, Sheikh Aziz (2004). Email consultations in health care: 2--acceptability and safe application. BMJ.

[19] Iafusco D, Ingenito N, Prisco F (2000). The chatline as a communication and educational tool in adolescents with insulin-dependent diabetes: preliminary observations. Diabetes Care.

[20] Zrebiec J F, Jacobson A M (2001). What attracts patients with diabetes to an internet support group? A 21-month longitudinal website study. Diabet Med.

[21] Eysenbach Gunther, Powell John, Englesakis Marina, Rizo Carlos, Stern Anita (2004). Health related virtual communities and electronic support groups: systematic review of the effects of online peer to peer interactions. BMJ.

[22] Esquivel Adol, Meric-Bernstam Funda, Bernstam Elmer V (2006). Accuracy and self correction of information received from an internet breast cancer list: content analysis. BMJ.

[23] Giménez-Pérez Gabriel, Gallach Maria, Acera Edita, Prieto Araceli, Carro Olga, Ortega Emilio, González-Clemente José-Miguel, Mauricio Dídac (2002). Evaluation of accessibility and use of new communication technologies in patients with type 1 diabetes mellitus. J Med Internet Res.

