# Supplementary material for: Stakeholder Perspectives on the Development of a Virtual Clinic for Diabetes Care: Qualitative Study
Source: J Med Internet Res. 2007 Aug 9;9(3):e23. doi: 10.2196/jmir.9.3.e23 (PMC2047282; doi:10.2196/jmir.9.3.e23)
Supplement: Supplementary file 1 [file jmir_v9i3e23_app1.ppt]

## Slide 1
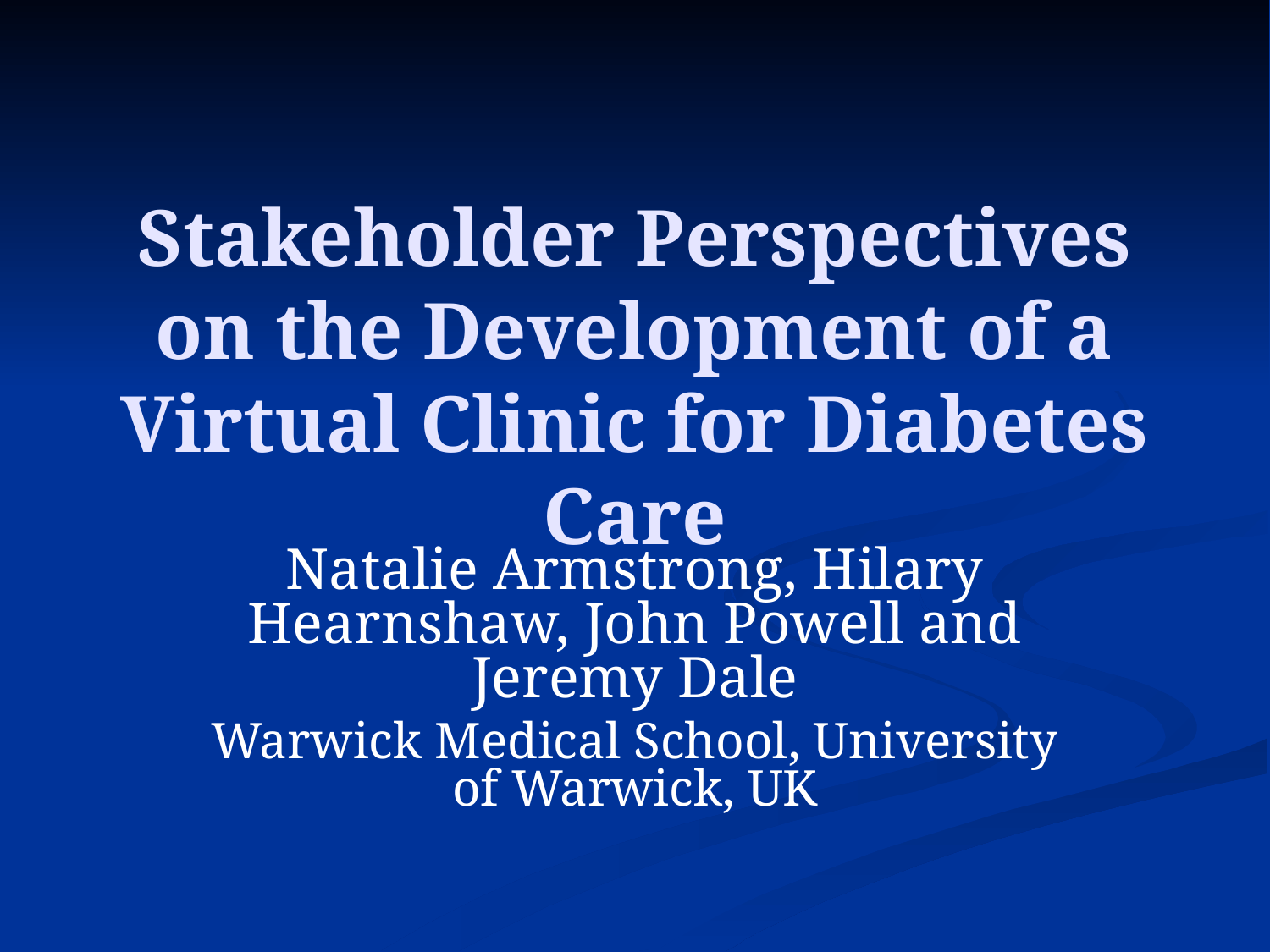

# Stakeholder Perspectives on the Development of a Virtual Clinic for Diabetes Care
Natalie Armstrong, Hilary Hearnshaw, John Powell and Jeremy Dale
Warwick Medical School, University of Warwick, UK

## Slide 2
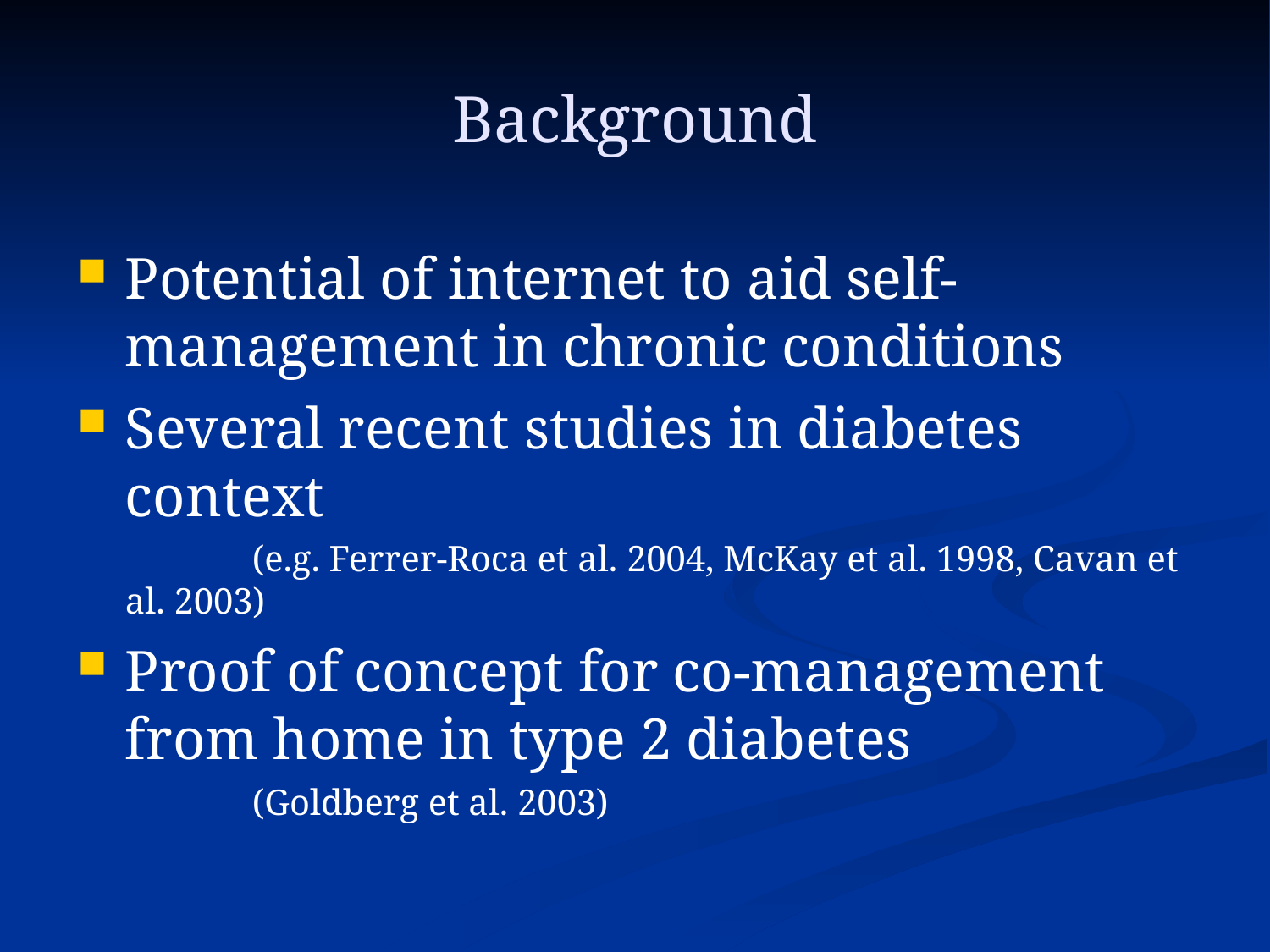

# Background
Potential of internet to aid self-management in chronic conditions
Several recent studies in diabetes context
	(e.g. Ferrer-Roca et al. 2004, McKay et al. 1998, Cavan et al. 2003)
Proof of concept for co-management from home in type 2 diabetes
	(Goldberg et al. 2003)

## Slide 3
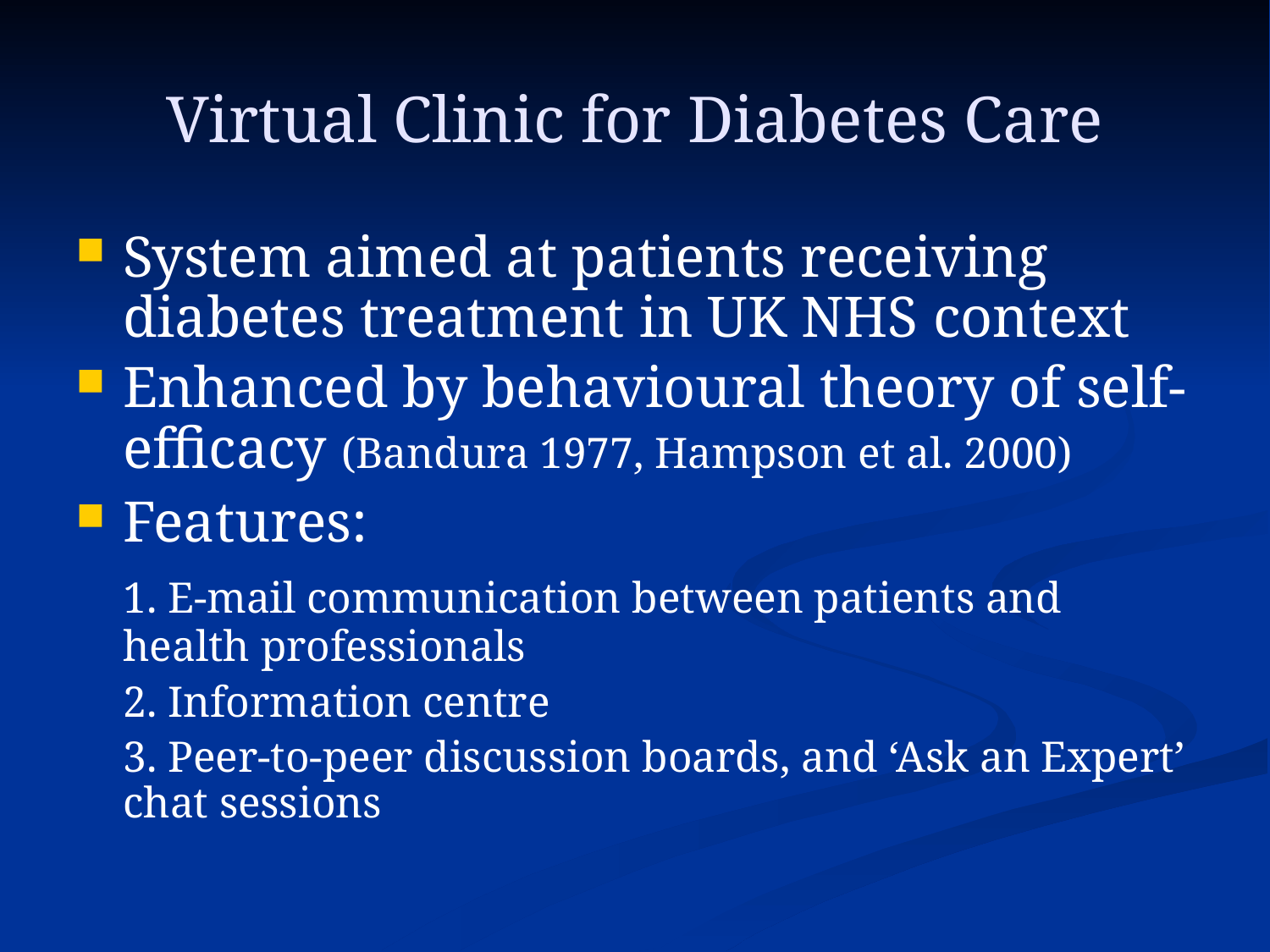

# Virtual Clinic for Diabetes Care
System aimed at patients receiving diabetes treatment in UK NHS context
Enhanced by behavioural theory of self-efficacy (Bandura 1977, Hampson et al. 2000)
Features:
	1. E-mail communication between patients and health professionals
	2. Information centre
	3. Peer-to-peer discussion boards, and ‘Ask an Expert’ chat sessions

## Slide 4
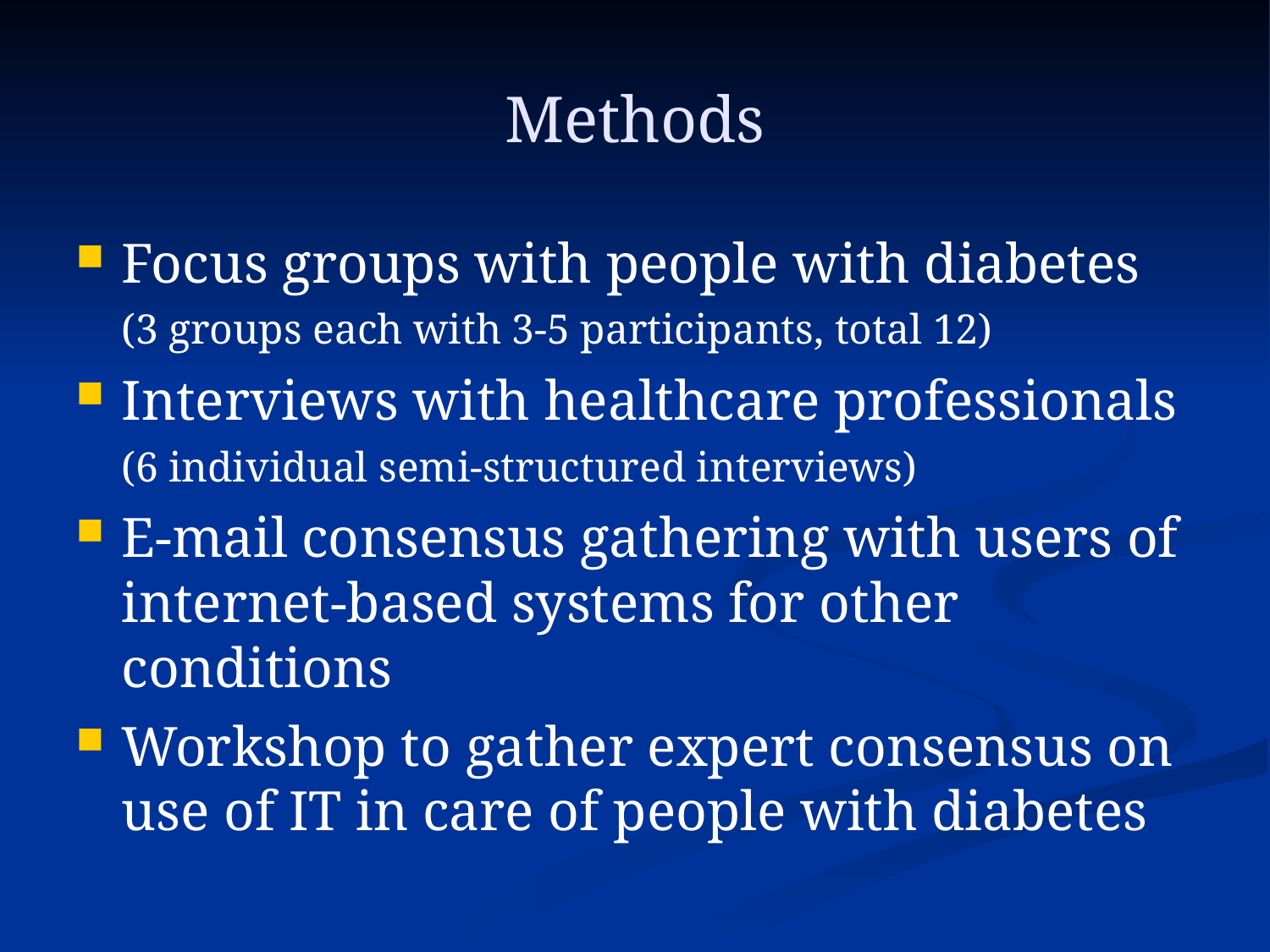

# Methods
Focus groups with people with diabetes
	(3 groups each with 3-5 participants, total 12)
Interviews with healthcare professionals
	(6 individual semi-structured interviews)
E-mail consensus gathering with users of internet-based systems for other conditions
Workshop to gather expert consensus on use of IT in care of people with diabetes

## Slide 5
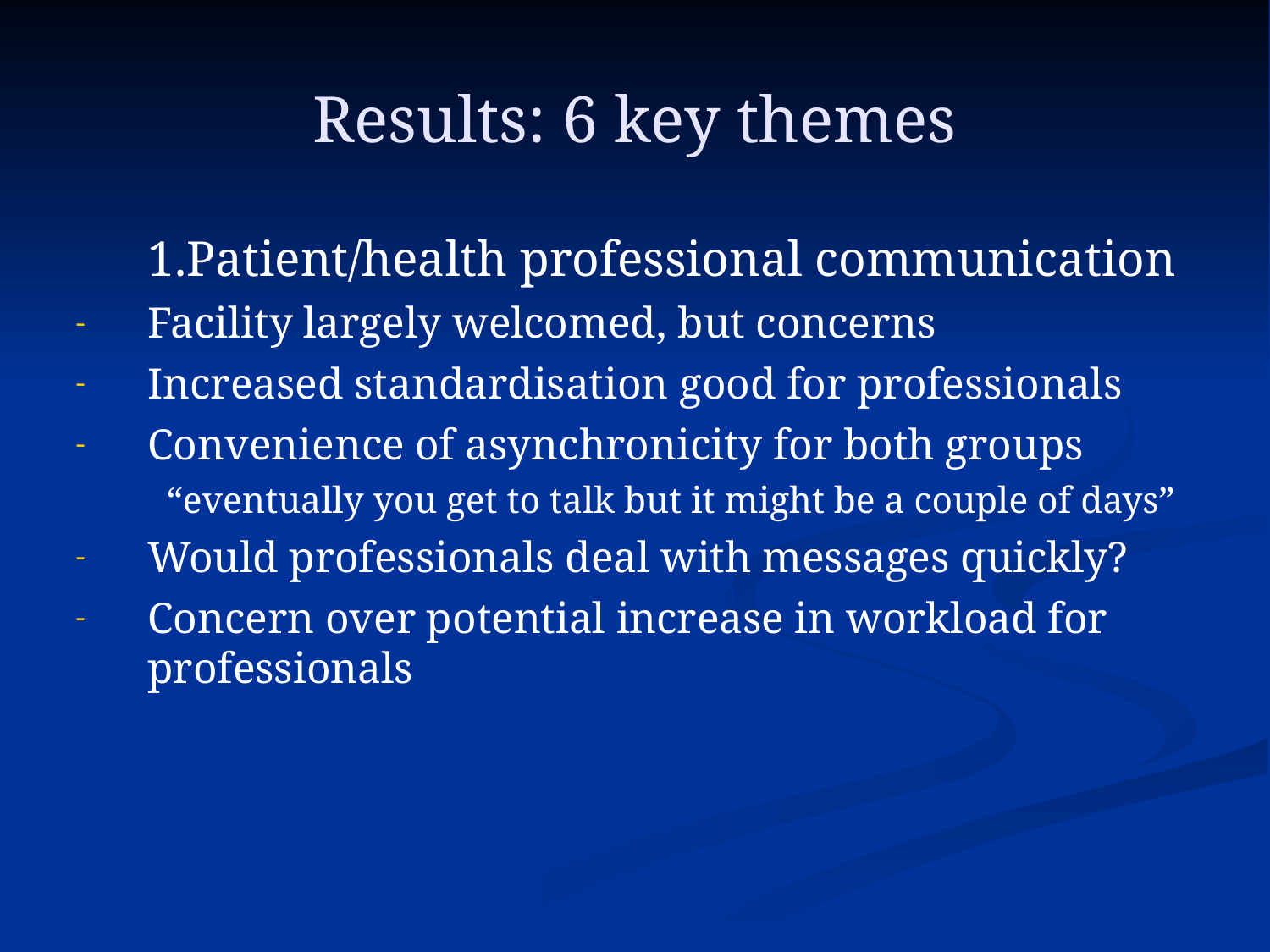

# Results: 6 key themes
1.Patient/health professional communication
Facility largely welcomed, but concerns
Increased standardisation good for professionals
Convenience of asynchronicity for both groups
	“eventually you get to talk but it might be a couple of days”
Would professionals deal with messages quickly?
Concern over potential increase in workload for professionals

## Slide 6
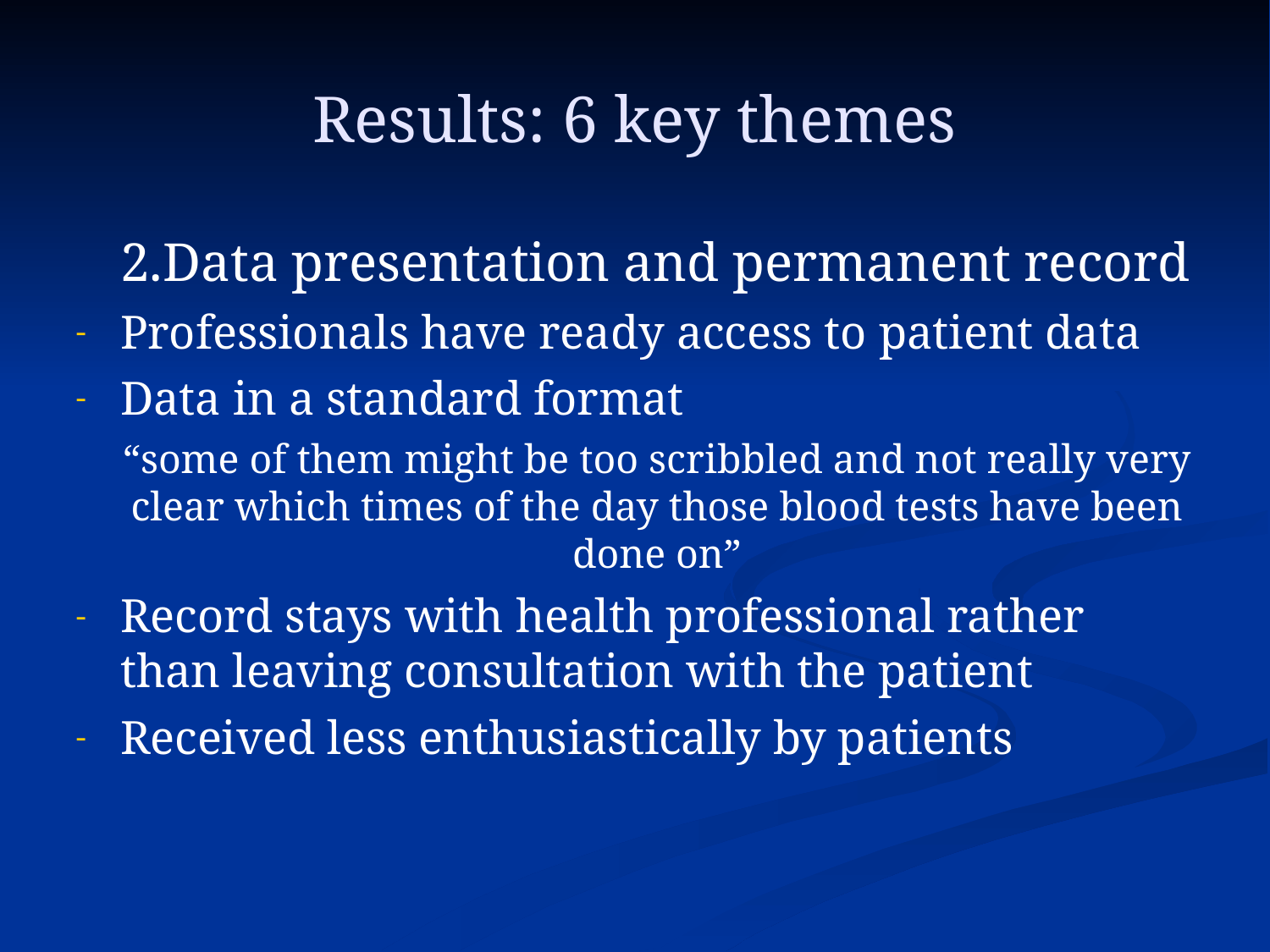

# Results: 6 key themes
2.Data presentation and permanent record
Professionals have ready access to patient data
Data in a standard format
	“some of them might be too scribbled and not really very clear which times of the day those blood tests have been done on”
Record stays with health professional rather than leaving consultation with the patient
Received less enthusiastically by patients

## Slide 7
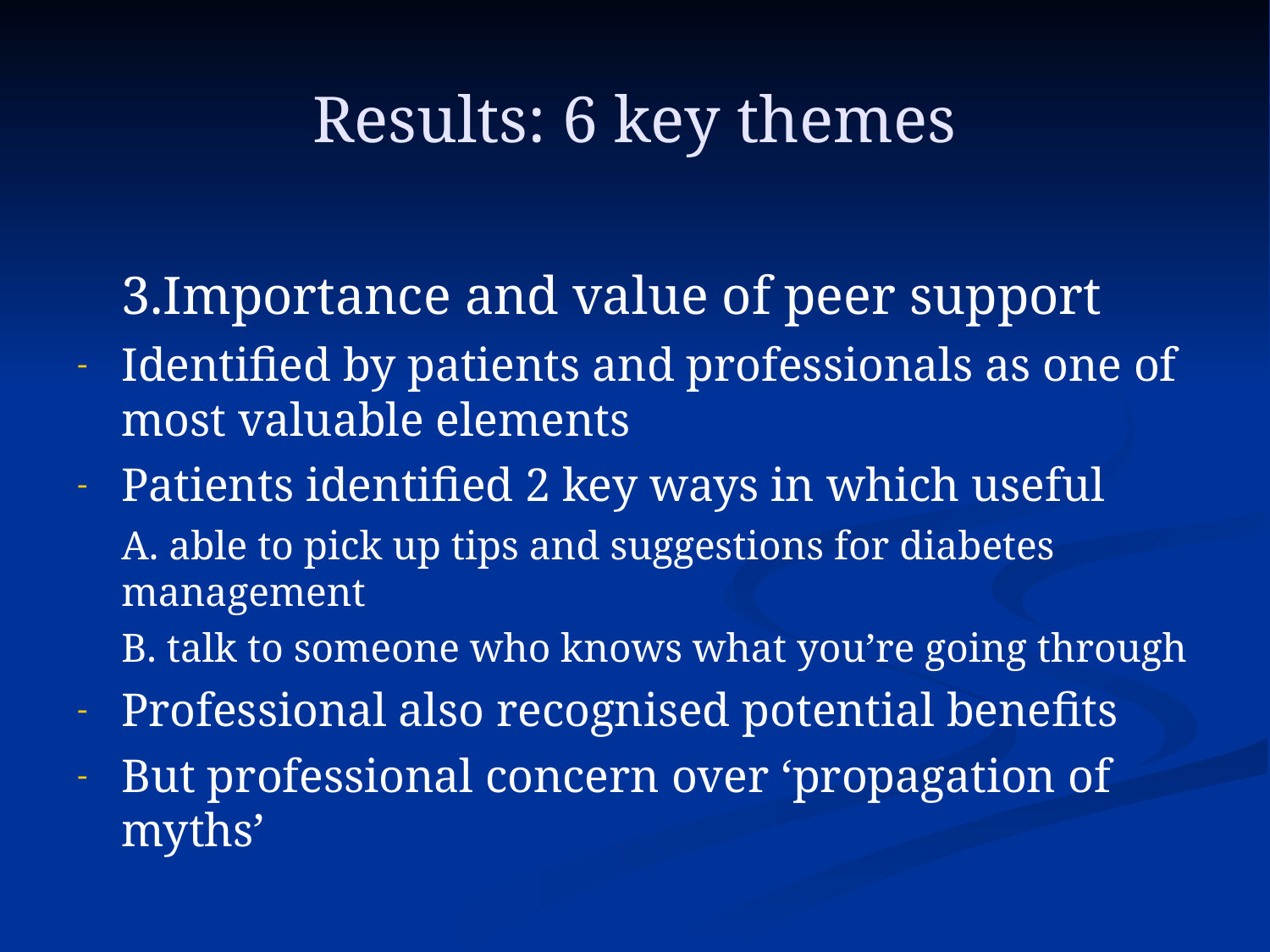

# Results: 6 key themes
3.Importance and value of peer support
Identified by patients and professionals as one of most valuable elements
Patients identified 2 key ways in which useful
	A. able to pick up tips and suggestions for diabetes management
	B. talk to someone who knows what you’re going through
Professional also recognised potential benefits
But professional concern over ‘propagation of myths’

## Slide 8
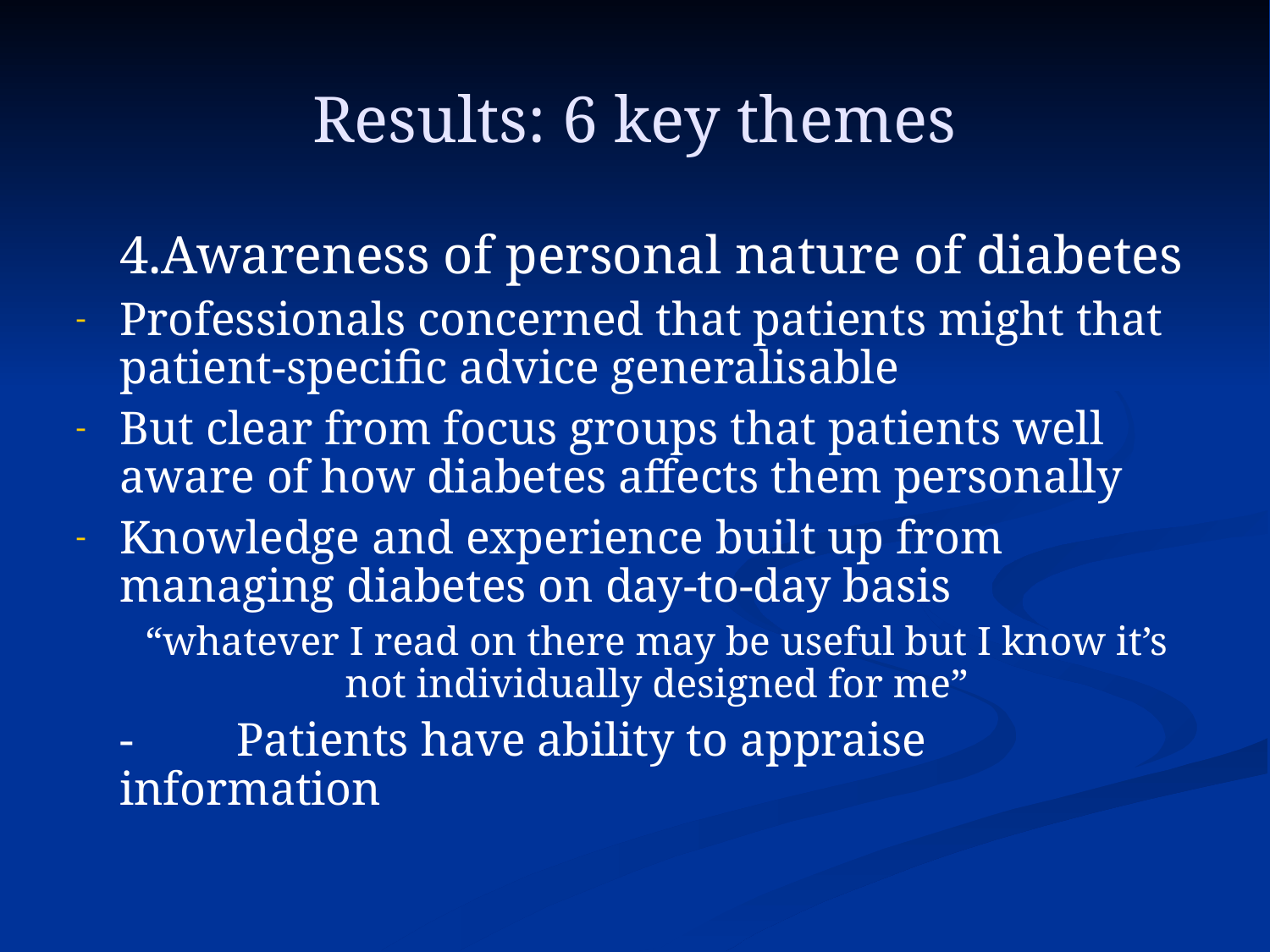

# Results: 6 key themes
4.Awareness of personal nature of diabetes
Professionals concerned that patients might that patient-specific advice generalisable
But clear from focus groups that patients well aware of how diabetes affects them personally
Knowledge and experience built up from managing diabetes on day-to-day basis
	“whatever I read on there may be useful but I know it’s not individually designed for me”
-	Patients have ability to appraise information

## Slide 9
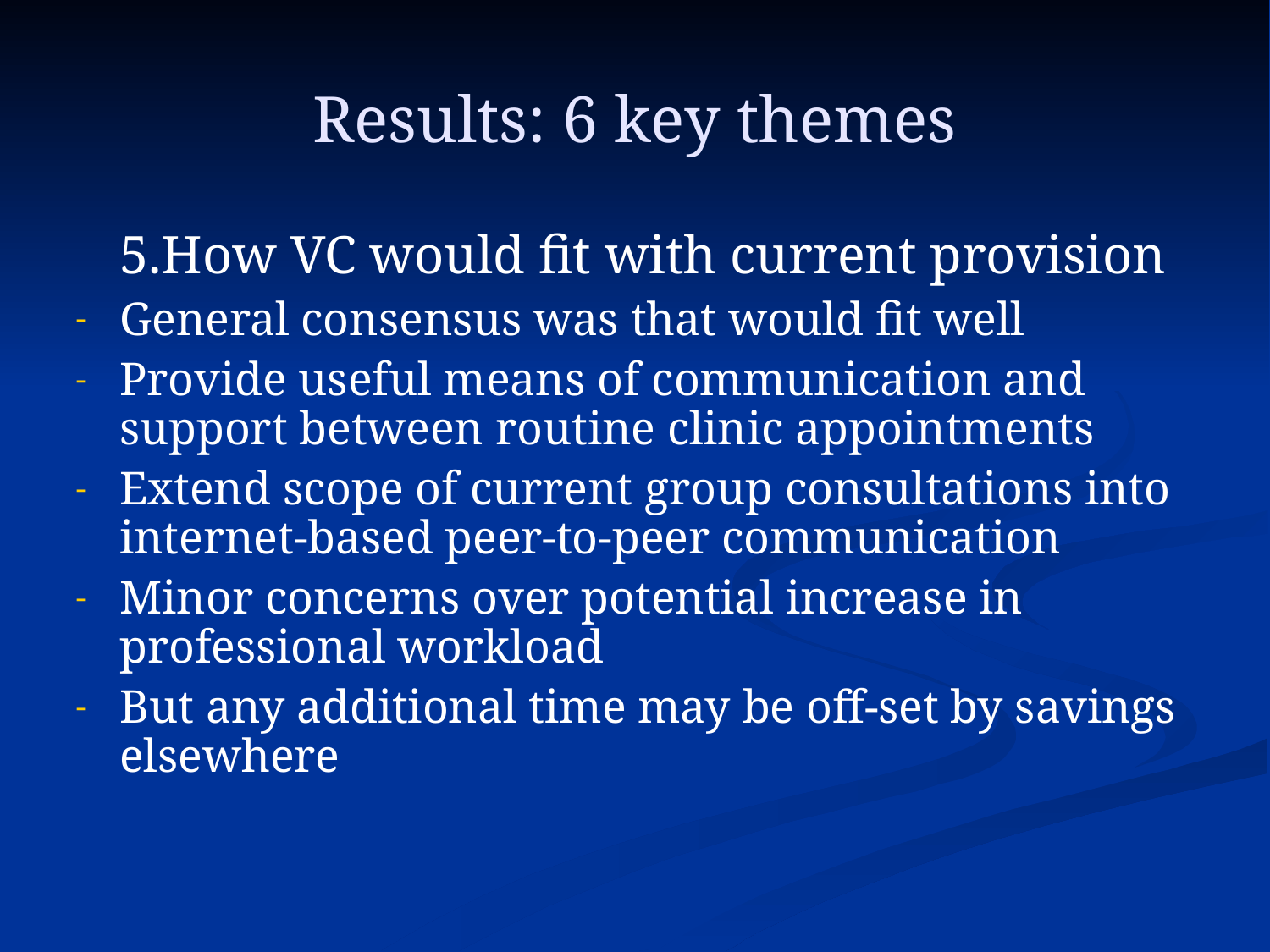

# Results: 6 key themes
5.How VC would fit with current provision
General consensus was that would fit well
Provide useful means of communication and support between routine clinic appointments
Extend scope of current group consultations into internet-based peer-to-peer communication
Minor concerns over potential increase in professional workload
But any additional time may be off-set by savings elsewhere

## Slide 10
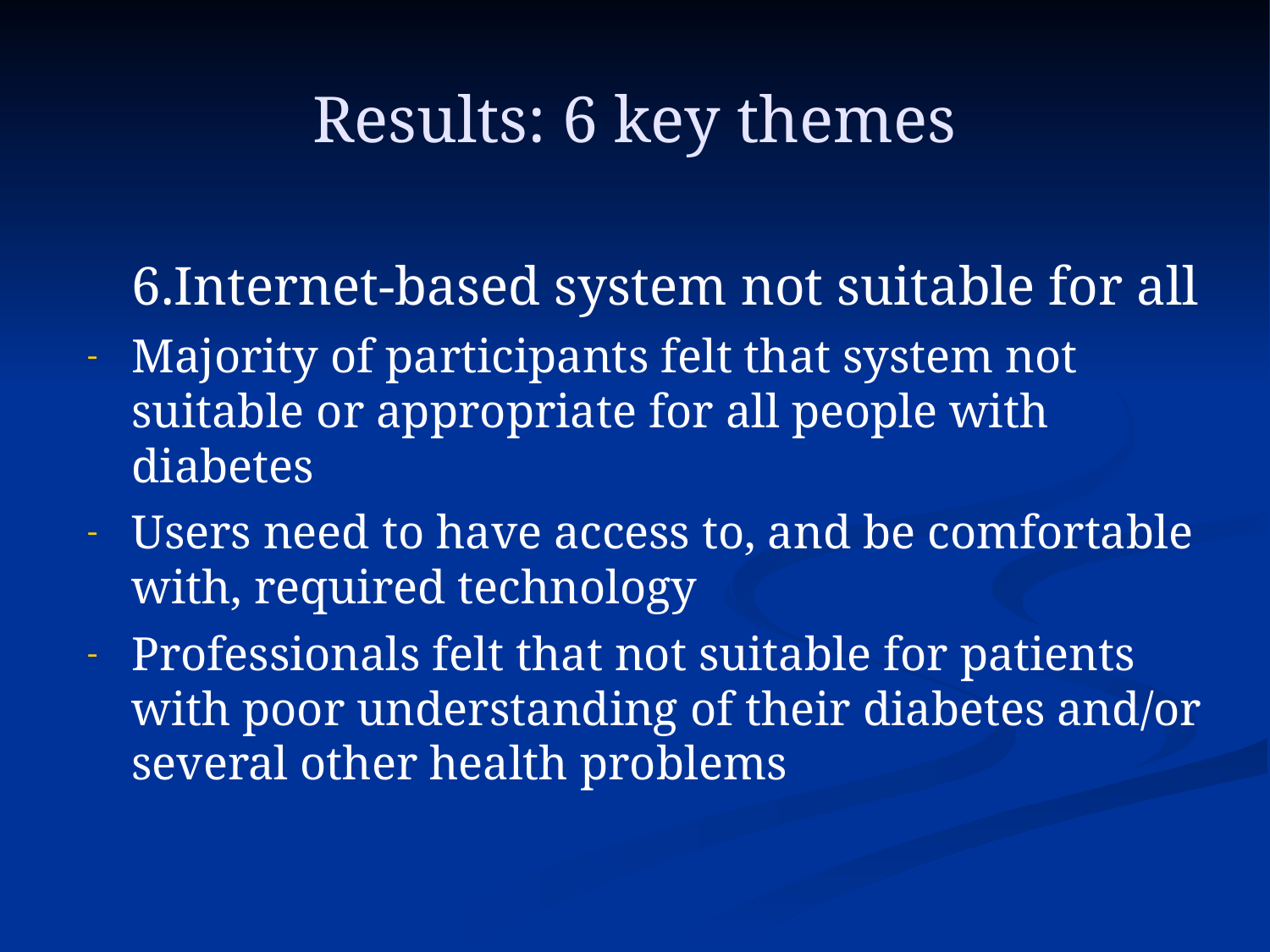

# Results: 6 key themes
6.Internet-based system not suitable for all
Majority of participants felt that system not suitable or appropriate for all people with diabetes
Users need to have access to, and be comfortable with, required technology
Professionals felt that not suitable for patients with poor understanding of their diabetes and/or several other health problems

## Slide 11
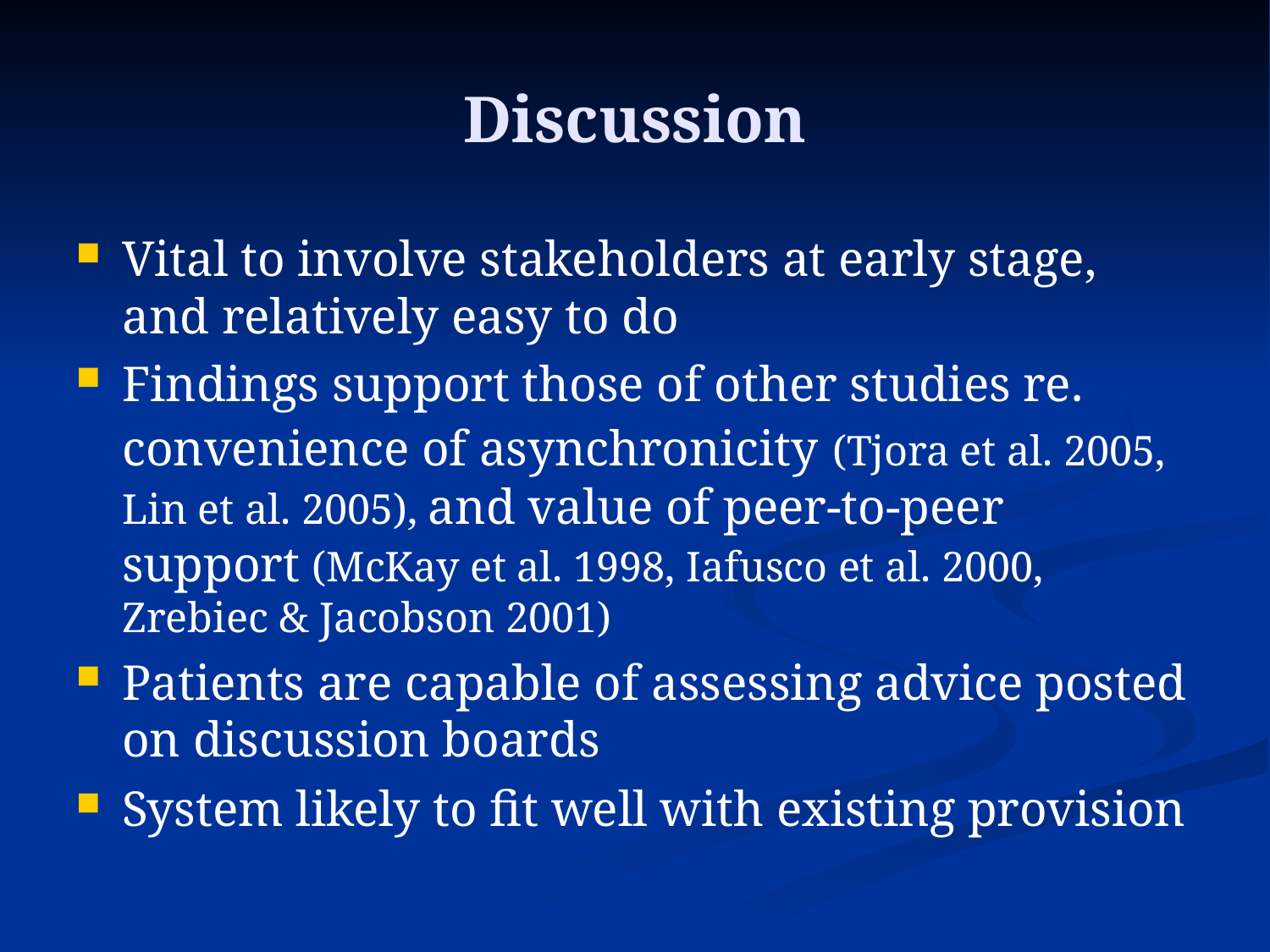

# Discussion
Vital to involve stakeholders at early stage, and relatively easy to do
Findings support those of other studies re. convenience of asynchronicity (Tjora et al. 2005, Lin et al. 2005), and value of peer-to-peer support (McKay et al. 1998, Iafusco et al. 2000, Zrebiec & Jacobson 2001)
Patients are capable of assessing advice posted on discussion boards
System likely to fit well with existing provision

## Slide 12
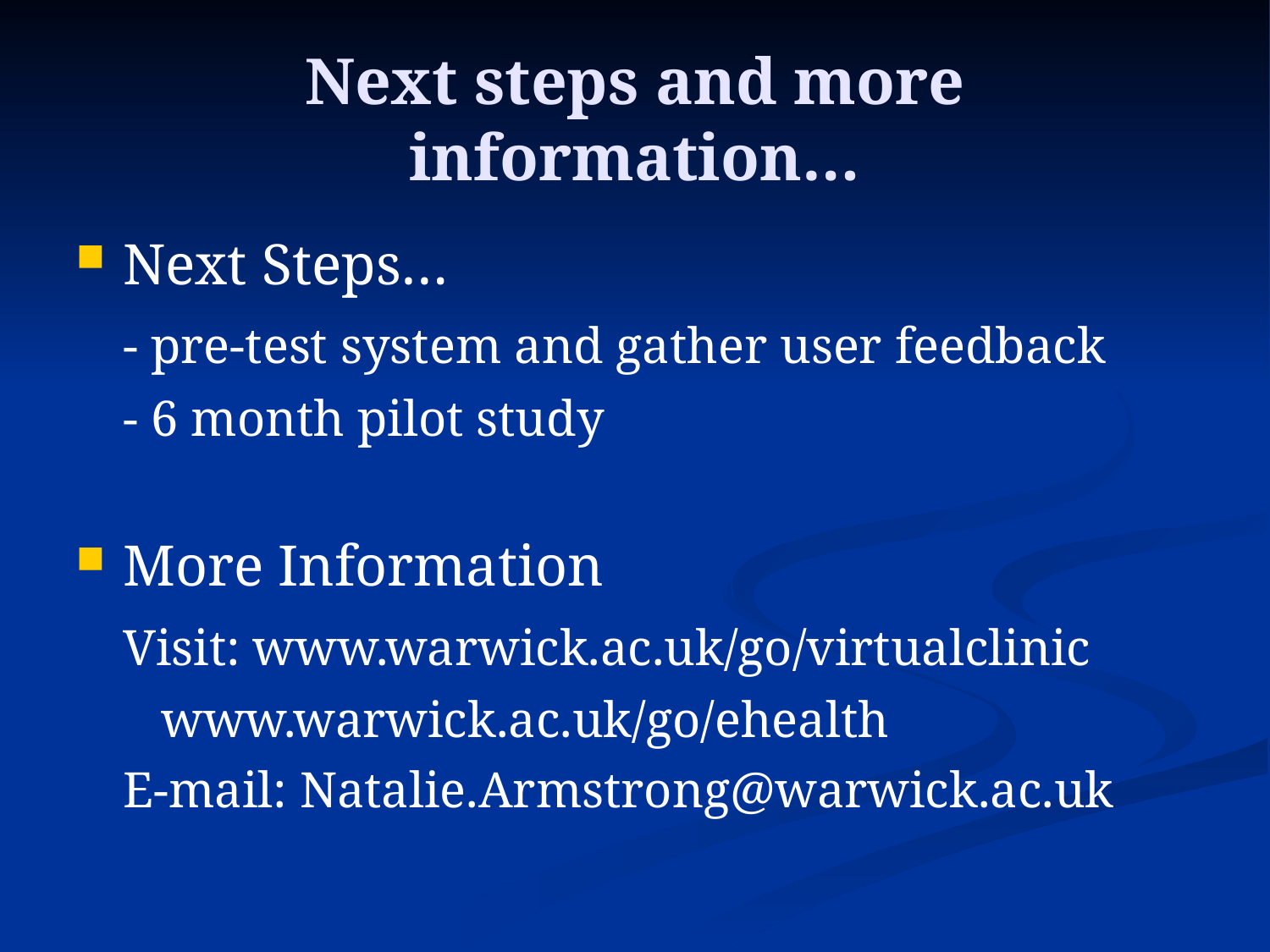

# Next steps and more information…
Next Steps…
	- pre-test system and gather user feedback
	- 6 month pilot study
More Information
	Visit: www.warwick.ac.uk/go/virtualclinic
		 www.warwick.ac.uk/go/ehealth
	E-mail: Natalie.Armstrong@warwick.ac.uk
